# Correlation Between Microscale Indentation Creep and Macroscale Tensile Creep of PLA/PCL Polymer Blends

**DOI:** 10.3390/ma19173783

**Published:** 2026-09-05

**Authors:** Adriana Vazquez-Pelayo, Veronika Gajdosova, Jiri Hodan, Miroslav Slouf

**Affiliations:** Institute of Macromolecular Chemistry, Czech Academy of Sciences, 16206 Prague, Czech Republic; pelayo@imc.cas.cz (A.V.-P.); gajdosova@imc.cas.cz (V.G.); hodan@imc.cas.cz (J.H.)

**Keywords:** microindentation, indentation creep, tensile creep, polymer blends, viscoelasticity

## Abstract

This study investigates the relationship between microscale indentation creep and macroscale tensile creep of immiscible polymer blends. Micro- and macroscale creep were measured across the full composition range of model poly(lactic acid)/poly(caprolactone) blends (PLA/PCL), using indentation loadings of 50 and 300 gf and a tensile loading of 2 kg. The creep data were fitted with the empirical power law (PL) model and three phenomenological elasto-visco-plastic (EVP) models implemented in our open-source Python package MCREEP (version 1.1.6). Traditional creep descriptors, such as indentation creep (*C*_IT_) defined by ISO standard or the creep exponent (*n*) from the well-established empirical PL model (deformation = *C*·*t^n^*), yielded inconsistent or even misleading results. However, detailed analysis of the creep curves showed that alternative creep descriptors, such as the total deformation corrected for the initial deformation, exhibited the same trends at the micro- and macroscale and strong linear correlations (*R*^2^ > 0.97). These findings demonstrate that short-term microindentation creep can predict the ranking of macroscale creep behavior in polymer blends, provided that suitable creep descriptors are employed.

## 1. Introduction

Creep is the time-dependent deformation of a solid material under a sustained load. It is particularly important in polymers due to their elasto-visco-plastic behavior, which is time-dependent and not always completely reversible [1,2]. Traditionally, creep of polymer materials is evaluated by macroscale tensile creep experiments conducted at relatively low stresses below the yield point [3]. The macro tensile creep tests require large samples and long experimental times, typically ranging from hours to days, weeks, or even months. Therefore, microindentation creep has emerged as an alternative to those demanding experiments. Compared to macroscopic tensile creep, microindentation creep involves higher localized stresses, significantly shorter testing times in the range of minutes, and much smaller samples [4,5].

In our previous work [5], several analyses were conducted to evaluate the potential of microindentation creep as a predictive tool for macroscopic tensile creep of individual polymers. It is worth noting that in our studies, the term *prediction* refers to the ability of microindentation creep to correctly rank different materials according to their macroscopic creep behavior, rather than to quantitatively predict the macroscopic creep response. A direct comparison between macroscale tensile creep and microscale indentation creep was performed using three common polymers: high-density polyethylene (PE; soft and ductile polymer), isotactic polypropylene (PP; harder material), and atactic polystyrene (PS; very hard and brittle). The results demonstrated that—for the individual polymers—the short-term microindentation experiments can predict the results of much longer macroscale tensile creep measurements at the qualitative level. In addition, an open-source Python package MCREEP (*https://pypi.org/project/mcreep*; accessed on 2 September 2026) was developed to process both tensile and indentation creep data in a consistent and efficient manner. The MCREEP package was described in the previous study [5]; it has been tested thoroughly, it comes with detailed documentation, and it is employed in the present study as well.

The main limitation of the previous work consisted in that the correlation between microscale indentation creep and macroscale tensile creep was investigated only for the simplest polymer systems, which are homopolymers. The fact that the simple, short microindentation creep measurements could predict the macroscale creep behavior of homopolymers was very encouraging, but it remained unclear whether the same approach is applicable to more complex polymer systems. To answer this question, we prepared poly(lactic acid)/poly(caprolactone) polymer blends (PLA/PCL) in the full composition range. The two polymers were selected not only due to their biodegradability and attractivity in recent research [6,7,8,9], but mostly for the very broad range of properties their blends can cover: from very soft and ductile PCL-rich blends to very stiff and brittle PLA-rich blends [10,11,12,13]. Previous studies have shown that the macroscopic tensile creep behavior of PLA-based materials is strongly influenced by secondary phases and interfacial interactions [14]. Although correlations between micro- and macroscopic mechanical properties have been reported for PLA/PCL blends [15,16,17], no studies have investigated the relationship between microindentation creep and macroscopic tensile creep in these materials.

Therefore, the aim of this study is to compare microindentation creep with macroscopic tensile creep in polymer blends. The work aims to establish a correlation between the micro- and macroscopic creep responses and to assess the reliability of microindentation as a predictive tool for the creep behavior of polymer systems. PLA/PCL blends were selected as the model system because they are of considerable current research interest and span a very broad range of mechanical properties, from the stiff and brittle PLA-rich compositions to the soft and ductile PCL-rich compositions.

## 2. Materials and Methods

### 2.1. Materials

Two biodegradable polymers were used in this study: poly(lactic acid) (PLA; NatureWorks, Plymouth, MN, USA, supplied by Resinex, Prague, Czech Republic; *T*_g_ ≈ 60 °C and *T_m_* ≈ 150 °C) and poly(ε-caprolactone) (PCL; Shenzhen Esun Industrial Co., Ltd., Nanshan District, Shenzhen, China; *T*_g_ ≈ −65 °C and *T_m_* ≈ 60 °C). For scanning-electron microscopy (SEM) sample preparation, tetrahydrofuran (THF; Lachner, Neratovice, Czech Republic) was used as an etching agent of the PCL phase, and sodium hydroxide (NaOH; Lachner, Neratovice, Czech Republic) was used for etching of the PLA phase.

### 2.2. Blend Preparation

PLA/PCL blends were prepared following a slightly modified version of the protocol described in our previous work [10]. In brief, PLA and PCL granulate were dried for 4 h at 80 °C and 12 h at 40 °C in a vacuum oven, respectively. The blends were prepared by melt-mixing in a laboratory kneader (Brabender Plasticorder, Brabender GmbH & Co.KG, Duisburg, Germany). Mixing was conducted at a rotation speed of 60 rpm for 8 min at 180 °C. After mixing, the material was compression-molded using two hydraulic presses (Fontijne Gortnes, Rotterdam, Netherlands). First, the material was hot-pressed at 190 °C for 2 min under a load of 50 kN, followed by 2 min at 100 kN. The sheets were then transferred to a second press and cooled with water to ambient temperature while maintaining a load of 100 kN. The prepared blends are summarized in Table 1.

### 2.3. Material’s Characterization

#### 2.3.1. Scanning Electron Microscopy

The morphology of PLA/PCL blends was analyzed using scanning electron microscopy (SEM). SEM micrographs were acquired with a MAIA3 microscope (TESCAN, Brno, Czech Republic). Before the SEM observations, all specimens were sputter-coated with Pt in a sputter coater (SCD 050; BAL-TEC AG, Balzers, Liechtenstein) to minimize sample damage and charging effects. Imaging was performed at an accelerating voltage of 3 kV in secondary electron mode.

Two sample preparations were employed to analyze the morphology [18,19]: (i) fracturing in liquid nitrogen to minimize plastic deformation, and (ii) cutting, followed by smoothing in liquid nitrogen as described elsewhere [18]. For the latter, following a previously reported methodology [19], PLA was etched in 20% NaOH at room temperature for 30 min, while PCL was removed by exposure to THF vapor at 45 °C for 5 min.

#### 2.3.2. Macromechanical Properties

Mechanical properties were characterized using a tensile testing machine (5582 universal testing machine; Instron, Norwood, MA, USA) equipped with a 1 kN load cell. Tensile tests were performed according to ISO 527-2 [20]. All tests were conducted at a controlled temperature of 22 °C.

#### 2.3.3. Microindentation and Microcreep

Microscale indentation creep measurements were carried using an instrumented microindentation hardness tester (MCT tester; CSM Instrument SA, Corcelles, Switzerland) with a Vickers indenter. In this method, a diamond square pyramid (with an angle of 136° between non-adjacent faces) is pressed into the surface of the specimens.

Smoothed surfaces for the microindentation experiments were prepared by means of cutting with a rotary microtome (RM 2155; Leica; Vienna, Austria), employing a commercial microtome blade for softer PCL-rich blends and a laboratory-made freshly broken glass knife (Leica EM KMR3; Leica, Vienna, Austria) for stiffer PLA-rich blends.

The experiments were performed at two different loading forces: *F* = 50 gf and 300 gf. For each specimen, at least 30 indentations were performed (using 3 cut surfaces prepared from 3 different locations of given sample and at least 10 indentations at each cut surface); the reported values correspond to the average of all indentations. Using the original software of the indentation device software (Indentation, version 9.0.16), whose algorithms are based on Oliver–Pharr theory [21], four micromechanical properties were determined from *F*–*h* or *h*–*t* curves according to the ISO 14577-1 standard [22], as previously described [23]: indentation modulus (*E*_IT_), indentation hardness (*H*_IT_), relative indentation creep (*C*_IT_), and elastic part of indentation work (*η*_IT_). Detailed definitions and calculation procedures for these micromechanical parameters are available in our previous publications [24,25]. Specifically, the indentation creep, *C*_IT_, as defined by the ISO 14577-1 standard [22], was calculated as:

(1) $$C_{\mathrm{IT}}=\frac{h_{2}-h_{1}}{h_{1}}\times 100\%$$

where *h*_1_ and *h*_2_ represent the indenter penetration depths measured at the beginning and at the end of the maximum-load holding segment, respectively.

#### 2.3.4. Macroscale Tensile Creep

Uniaxial tensile creep was measured following the previously described, well-established methodology [26], which has been shown suitable for polymer systems [5,26,27]. The measuring device was a laboratory-built apparatus consisting of a dead weight attached to a mechanical stress amplifier (lever ratio 10:1). A digital displacement gauge was connected to the upper specimen clamp to record the displacement and transfer the data to a PC [5].

The specimens were prepared by machining of compression-molded sheets into dumbbell-shaped specimens according to ISO 527-2 [20]. The specimen cross-section was 4 mm × 10 mm, and the initial gauge length was 100 mm. Before testing, all specimens were stored in a dark room at a stable temperature (~22 °C).

Mechanical pre-conditioning was applied before each creep measurement and consisted of a one-minute application of stress, producing a strain higher than the expected final strain reached during the subsequent creep experiment. This was achieved by applying twice the loading used in the subsequent creep experiment. The suitability of this 2× higher loading was documented experimentally by the recorded creep curves and is also justified theoretically by the fact that doubling the applied load produces twice the initial deformation, while the subsequent creep deformation is substantially lower for common polymer systems, including PLA/PCL blends. A recovery period of at least 1 h was allowed after pre-conditioning.

For each blend, a series of tensile creep measurements with a hold time of 100 min and loadings up to one half of the estimated yield stress was performed. The yield stress could be estimated either using a simple linear model or using the more sophisticated equivalent box model, as described elsewhere [10]. The fact that all loadings were below one half of the yield stress guaranteed that the short-term pre-conditioning experiments (which were performed with double loading) did not result in significant permanent plastic deformation of the tested specimens. For all samples, the loadings up to 2 kg (corresponding to a stress of 4.91 MPa) were safely within linear viscoelastic regime (LVE, where the deformation was proportional to the applied stress within the experimental error). All creep measurements were conducted in an air-conditioned room at 22 °C. To mitigate possible local temperature variations, the creep devices were placed inside a closed wooden box lined with 2 cm of foamed polystyrene on all sides (including the doors).

In this work, we employed and evaluated only the creep measurements performed at a loading of 2 kg (stress of 4.91 MPa). The selected loading was sufficiently low for the softest PLA/PCL (0/100) samples (yield stress of PCL ≈ 16 MPa) and sufficiently high to produce detectable creep in the stiffest PLA/PCL (100/0) samples (yield stress of PLA ≈ 50 MPa). The identical loading and experimental conditions for all blends ensured that the creep descriptors, including the total creep deformation, were directly comparable. All creep measurements were conducted at least twice. If the two creep curves differed by more than 20%, another two measurements were performed. Therefore, the final tensile creep curves in this study are averages of at least two measurements.

### 2.4. Creep Data Evaluation

MCREEP package [5] was used for fitting creep models to experimental data. The package supports both tensile and indentation creep analyses. The creep data can be fitted using the empirical power law model (PL; Equations (1) and (2)) and phenomenological elasto-visco-plastic models (EVP; Equations (3) and (4)). MCREEP is an open-source software, whose documentation, installation instructions, and worked examples are available in its official repository (*https://pypi.org/project/mcreep*). The PL models implemented in MCREEP package for tensile creep (Equation (1)) and indentation creep (Equation (2)) measurement are:

(2) $$\varepsilon \left(t\right)=C\cdot t^{n}$$

(3) $$h{\left(t\right)}^{2}=C\cdot t^{n}$$

where *ε*(*t*) is the tensile strain, h(t) is the indentation penetration depth, *C* and *n* are empirical material constants, and *t* is the creep time. It is worth noting that the creep coefficient, *C*, and creep exponent, *n*, from PL usually take different values in the case of tensile and indentation experiments. The EVP models implemented in MCREEP package for tensile (Equation (4)) and indentation creep (Equation (5)) measurements are:

(4) $$\varepsilon \left(t\right)=\frac{\sigma }{E_{0}}+\sigma \frac{t}{\eta }+\sigma \sum_{i=1}^{N}\frac{1}{E_{i}}\left[1-exp\left(-\frac{t}{\tau_{i}}\right)\right]$$

(5) $${h\left(t\right)}^{2}=FK\left[B+C_{\upsilon }t-\sum_{i=1}^{N}D_{i}\;\exp\left(-\frac{t}{\tau_{i}}\right)\right]$$

where, in Equation (4), *σ* is the applied stress, *E_0_* is the instantaneous elastic modulus, *η* is a parameter related to viscous flow, *E_i_* is a modulus of the *i*-th viscoelastic Kelvin–Voigt element (KV; as described elsewhere [28,29]), and *τ_i_* is a retardation time of the corresponding *i*-th element; in Equation (5), *F* is the applied load, *K* is the geometric constant of the Vickers indenter, and *B*, *C_ν_*, *D_i_*, and *τ_i_* are regression parameters obtained by fitting the indentation creep data, analogous to their counterparts in Equation (4). Depending on the number of viscoelastic elements included in the model, three EVP variants (S + D + 1 KV, S + D + 2 KV, and S + D + 3 KV) were evaluated, corresponding to one, two, and three viscoelastic KV terms, respectively. Increasing the number of viscoelastic terms enables the model to capture creep responses with progressively greater accuracy. Detailed explanation of all models and parameters can be found in our previous study [5].

## 3. Results

### 3.1. Basic Characterization of the Investigated PLA/PCL Blends

#### 3.1.1. Morphology

**Figure 1:**
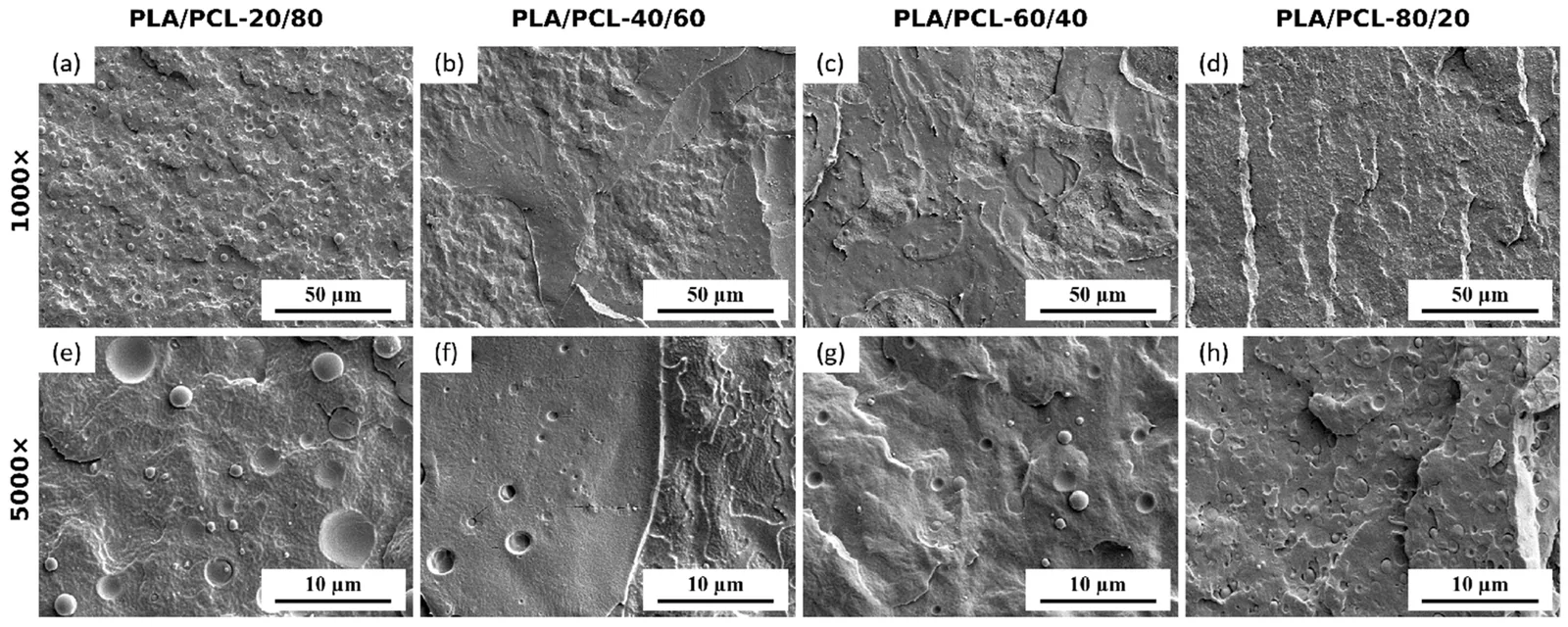
SEM micrographs showing fracture surfaces of PLA/PCL blends at lower (**a**–**d**) and higher (**e**–**h**) magnification.

Phase morphology of the PLA/PCL blends (Figure 1) follows general trends [30]. At low PLA content (PLA/PCL-20/80), PCL forms the continuous matrix, while PLA is present as a finely dispersed droplet phase with relatively uniform size and no evident agglomeration (Figure 1a,e). The fracture surface at this composition displays microplastic deformations, typical of the ductile PCL matrix. As the PLA fraction increases (PLA/PCL-40/60), the dispersed PLA domains grow in size and coalesce, leading to a progressive coarsening of the phase structure, partial co-continuity, and regions of phase inversions, where PLA domains contain PCL particles (Figure 1b,c,f,g). Finally, at the highest PLA concentration (PLA/PCL-80/20), a complete inversion of morphology occurs, with PLA forming the continuous matrix and PCL appearing as the dispersed phase (Figure 1d,h). The fracture surface here exhibits sharp fracture lines and quasi-brittle cracks, indicative of the higher stiffness, brittleness, and restricted molecular mobility of the PLA-dominant matrix.

**Figure 2:**
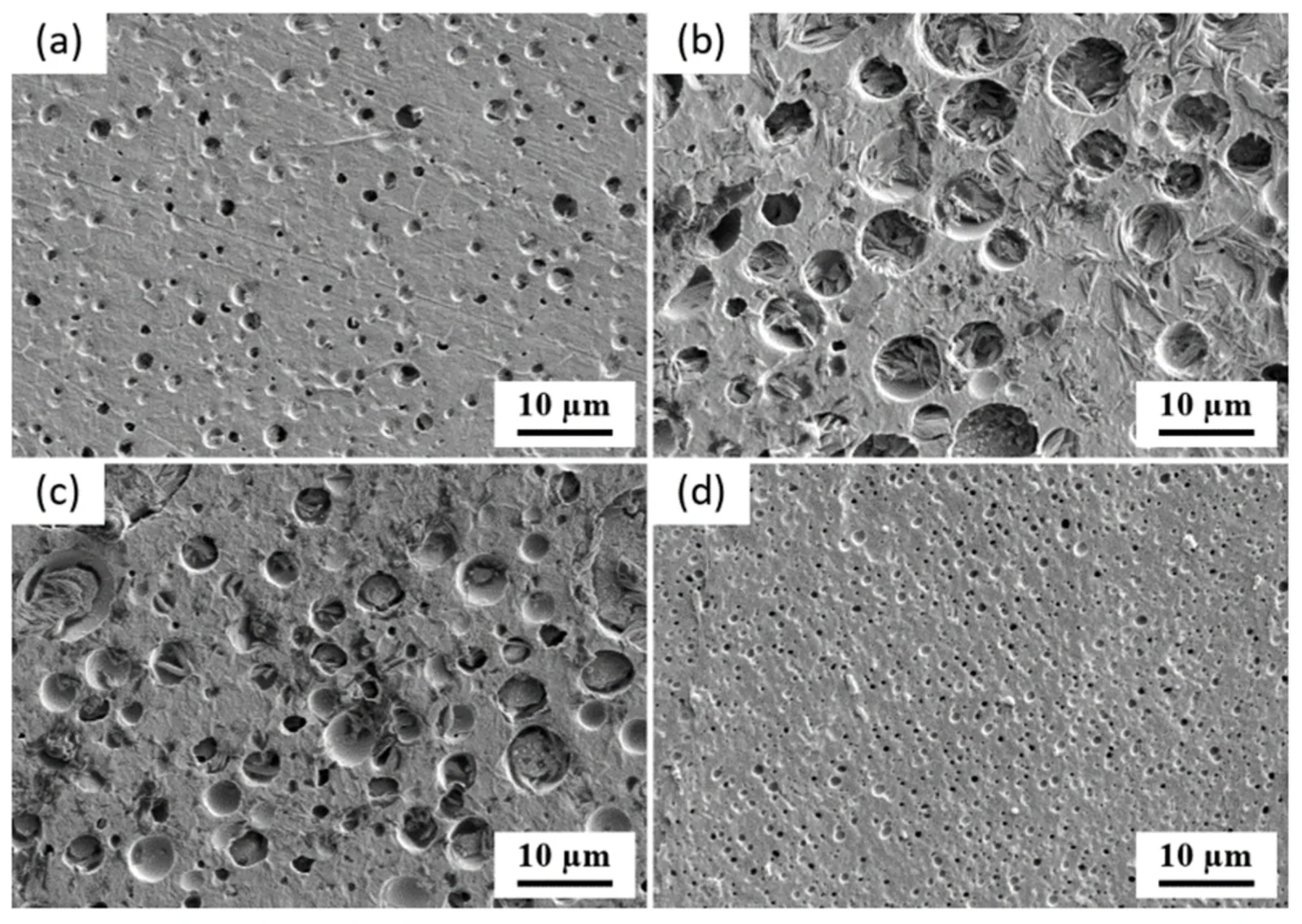
SEM micrographs showing smoothed and etched surfaces of PLA/PCL blends: (**a**) PLA/PCL-20/80, (**b**) PLA/PCL-40/60, (**c**) PLA/PCL-60/40, and (**d**) PLA/PCL-80/20.

The smoothed and etched PLA/PCL blend surfaces (Figure 2) enabled a more detailed visualization of the phase morphology. The PLA/PCL-20/80 and PLA/PCL-80/20 blends exhibited a droplet–matrix morphology. The micrographs confirmed a markedly coarser morphology near the 50/50 composition, consistent with increased phase connectivity. The selected micrographs of PLA/PCL-40/60 and PLA/PCL-60/40 in Figure 2 highlight regions with particulate morphology, whereas phase co-continuity is more evident in the lower-magnification SEM fracture surface micrographs in Figure 1.

#### 3.1.2. Micro- and Macromechanical Properties

**Figure 3:**
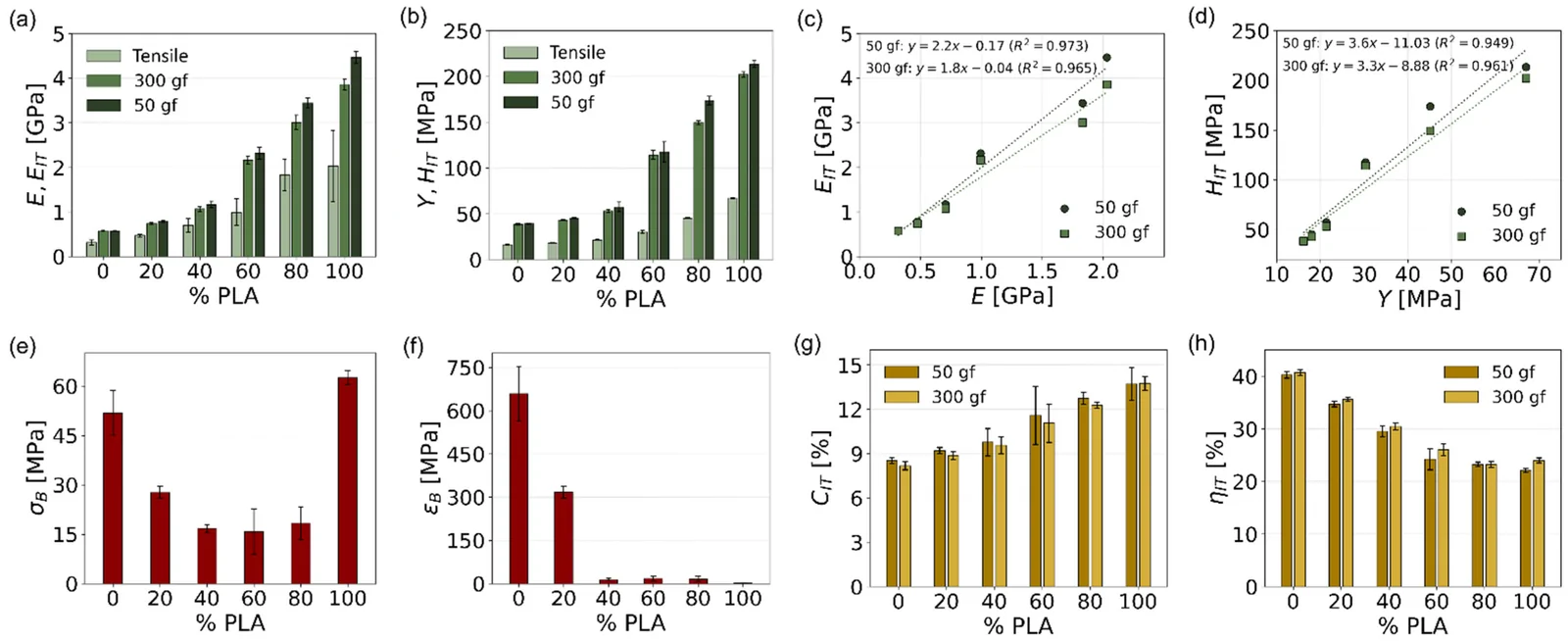
Micro- and macromechanical properties: (**a**) Tensile modulus (*E*) and indentation modulus (*E*_IT_), (**b**) yield strength (*Y*) and indentation hardness (*H*_IT_), (**c**) correlation between *E* and *E*_IT_, (**d**) correlation between *Y* and *H*_IT_, (**e**) stress at break (*σ*_B_), (**f**) strain at break (*ε*_B_), (**g**) indentation creep (*C*_IT_), and (**h**) elastic part of indentation work (*η*_IT_) determined according to ISO 14577-1 [22].

Figure 3 summarizes the mechanical response of the PLA/PCL blends, integrating both micro- and macromechanical properties. The figure comprises stiffness-related properties at both scales (Figure 3a–d), ultimate properties from macroscale tensile testing (Figure 3e,f), and viscosity-related properties from microindentation testing (Figure 3e–h).

All stiffness-related properties (Figure 3a,b) exhibited the expected monotonic increase with PLA content, reflecting the gradual transition from soft PCL (*T*_g_ ≈ −65 °C; ductile polymer at room temperature) to stiff PLA (*T*_g_ ≈ 60 °C; hard and brittle polymer at room temperature). The linear correlation of the microscale indentation moduli with the macroscale tensile modulus (*E*_IT_ ∝ E; Figure 3c) is in line with Oliver–Pharr theory [31,32]. The linear increase of microscale indentation hardness with the macroscale yield stress accords with Tabor’s relation (*H*_IT_ ≈ 3*Y*; ref. [33]). The observed correlations between micro- and macroscale properties confirmed the reliability and correctness of our micromechanical measurements. The standard deviations for microindentation measurements were reasonably low in the whole concentration range, which documented both the precision of the measurements and the fact that the indents were large enough to probe the whole blend rather than its individual phases. The indent size for the roughest PLA/PCL blends with composition 40/60 and 60/40 was >90 μm, which was well above the characteristic dimensions of structural inhomogeneities observed in SEM (Figure 1 and Figure 2). This can be verified using the approximate formula *H*_IT_ ≈ 1.854⋅*F*/*d*^2^, where *F* is the maximum force (50 or 300 gf) and *d* is the diagonal length of the indent on the polymer surface [34].

The ultimate properties from tensile testing (Figure 3e,f) and viscosity-related properties from indentation measurements (Figure 3g,h) exhibited a more complex behavior. The stress at break (*σ*_B_; Figure 3e) was high for ductile PCL-rich blends and stiff PLA-rich systems, while the intermediate compositions showed a marked reduction of *σ*_B_. Similar non-linear behavior is often observed for immiscible polymer blends [8,19]. The elongation at break (ε_B_; Figure 3f) exhibited a steep decrease for the systems with continuous or partially continuous PLA matrix due to its brittleness. Indentation creep calculated according the ISO standard (*C*_IT_; Figure 3g) showed a counter-intuitive increase with PLA concentration, as if the stiffer, PLA-rich blends exhibited higher deformation and lower resistance to long-term loading than the softer, PCL-rich blends. This suggested that *C*_IT_ was not a suitable parameter to characterize the creep of polymer blends. Similar counter-intuitive behavior was observed for the same ISO-standard-defined elastic part of the indentation work (*η*_IT_; Figure 3h), which decreased with the increasing PLA concentration, as if the stiffer PLA-rich blends exhibited lower elasticity than the ductile PCL-rich blends. This seemingly paradoxical behavior is connected with the fact that the elasto-visco-plastic properties of PLA/PCL blends change in broader range than typical for common inorganic materials, as explained in Appendix A.

To summarize, the correlations between stiffness-related properties at both micro- and macroscale (Figure 3a–d) confirmed the capability of indentation techniques to capture bulk mechanical performance of polymer systems [35]. The ultimate properties from tensile testing (Figure 3e,f) exhibited non-linear but logical trends in agreement with other similar studies [19]. The counter-intuitive trends observed for viscosity-related properties from indentation testing (Figure 3g,h) illustrated certain limitations of the ISO 14577-1 standard [22], which was developed for the instrumented indentation testing of inorganic materials, and indicated that proper description of the creep behavior of polymer systems requires more advanced methods.

### 3.2. Microscale Indentation Creep

**Figure 4:**
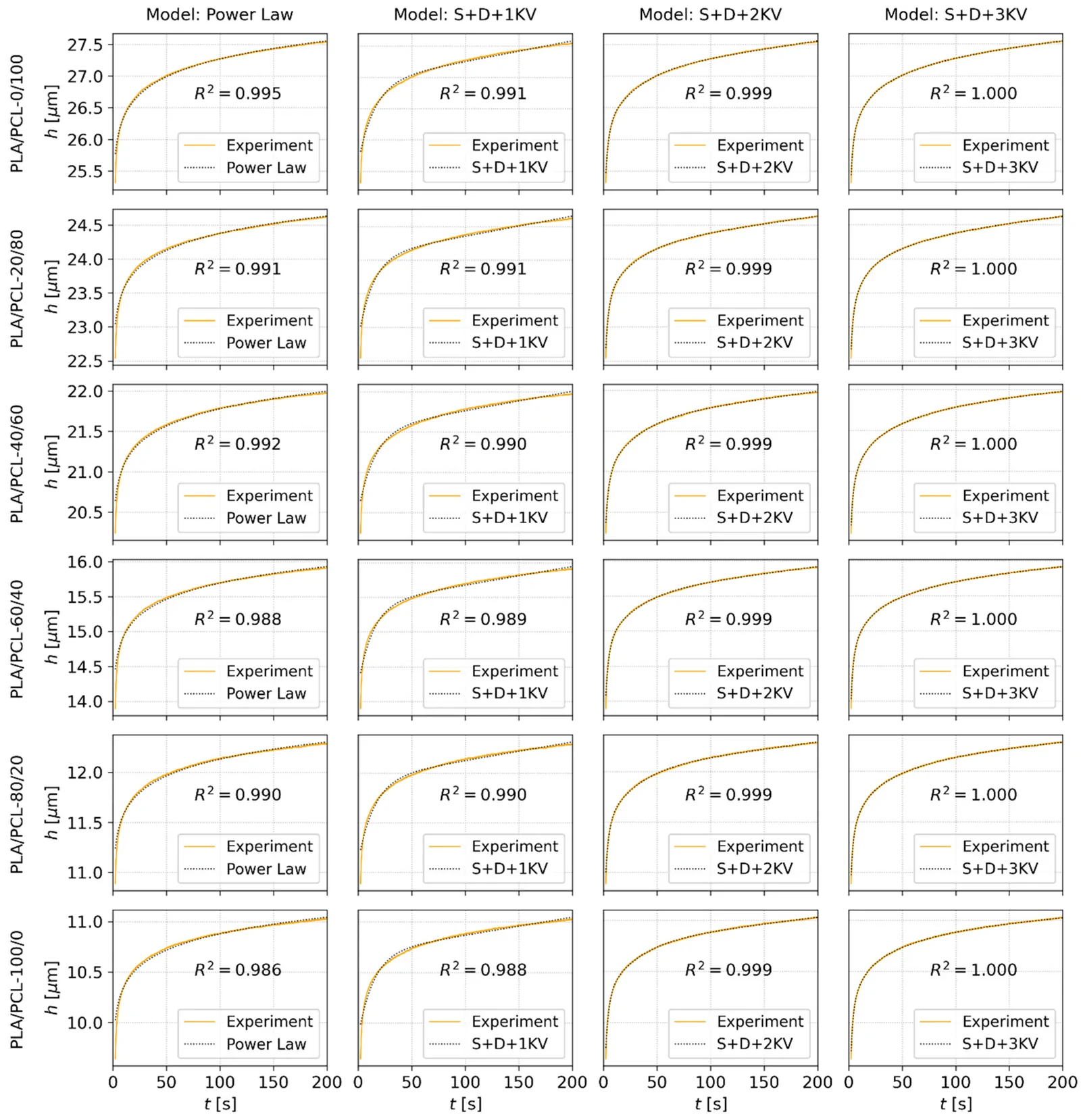
Fitting of creep predictive models to microscale indentation creep data measured at *F* = 50 gf for all investigated PLA/PCL blends. The columns show, from left to right, fitting with PL model and EVP models with 1, 2, and 3 KV elements, respectively. Each plot presents the coefficient of determination, *R*^2^, as a measure of fit quality, with *R*^2^ = 1.0 corresponding to a perfect fit. Each indentation creep measurement was repeated 15 times, but the figure displays just one representative curve from each sample–model combination.

Figure 4 shows the fitting of creep predictive models to experimental *h*–*t* curves obtained under a load of 50 gf. Although the analyses were performed at two loading forces (50 gf and 300 gf), only the 50 gf data are shown here for the sake of brevity. The 300 gf data showed very analogous trends. The final averaged fitting results of all measurements at both loads are summarized in Appendix B.

The PL and three EVP models (described in the Experimental section) were fitted to the experimental data over the entire time range (0–200 s). For this relatively short time interval, all models could fit the experimental data very well. The simple PL model (Equation (3)) yielded a good overall fit, although small deviations could be observed for short times. The reason consists in that the empirical PL model was designed to predict long-term creep behavior, rather than the initial creep stage. The EVP models showed good agreement with the experimental data for all compositions, capturing both the initial and long-term time-dependent deformation. As the number of fitting parameters increases, the EVP models fit the experimental data more accurately (as evidenced by the increase of *R*^2^), but the fitting is less stable (as the models can be overfitted for a given dataset [4]). The fit stability for EVP models could be improved by fixing the retardation times (*τ*_i_ in Equation (5)) at suitable pre-fitted values, as discussed in detail elsewhere [4,5] and summarized briefly for our case of PLA/PCL blends in Appendix B.

### 3.3. Macroscale Tensile Creep

**Figure 5:**
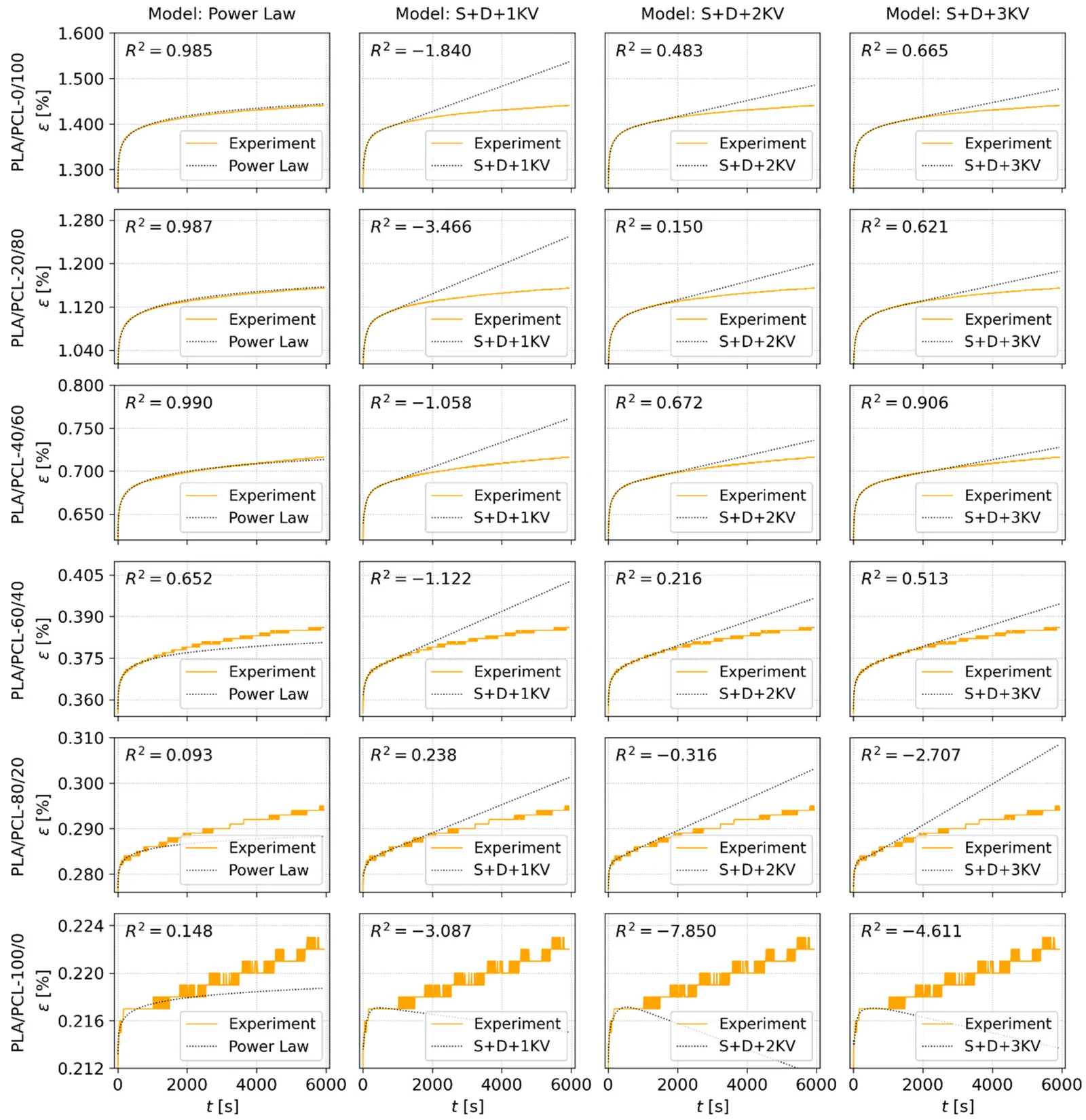
Fitting of creep predictive models to macro tensile creep data measured at 2 kg for all investigated PLA/PCL blends. The columns show, from left to right, fitting with PL model and EVP models with 1, 2, and 3 KV elements, respectively. Each plot presents the coefficient of determination, *R*^2^, as a measure of fit quality, with *R*^2^ = 1.0 corresponding to a perfect fit. The figure displays the averaged *ε*–*t* curves.

Figure 5 shows the fitting of the creep prediction models to the *ε*–*t* curves obtained from macroscale tensile creep tests for all investigated PLA/PCL blends. Each tensile creep experiment was performed at least twice, and the results were averaged. If the two measurements differed substantially, the experiment was repeated, and the final creep curve was obtained by averaging all measurements. Figure 5 displays the averaged *ε*–*t* curves. The final fitting results for all measurements are summarized in Appendix C.

Unlike the microscale indentation creep analysis, where the models were fitted over the entire holding period (0–200 s), the PL and EVP models were fitted only to the first 1000 s of the tensile creep data. This fitting strategy was adopted to evaluate the models’ long-term predictive capability by comparing their predictions, based on the initial 0–1000 s of the experiment, with the complete experimental creep response over the full 0–6000 s period.

The empirical PL model provided a good overall fit for the PCL-rich blends, yielding a satisfactory description of the long-term viscoelastic response of these softer compositions (*R*^2^ > 0.98 for all blends up to 40% of PLA). In contrast, larger deviations were observed for the PLA-rich blends. Owing to their higher stiffness, these materials exhibited limited tensile creep deformation, resulting in stepwise strain increments and consequently greater experimental scatter (Figure 5, lower row). Moreover, the PLA-rich blends exhibited a secondary creep regime, characterized by a linear increase in strain following the transient stage. This behavior could not be accurately reproduced by the intrinsically non-linear PL model, resulting in lower prediction accuracy and reduced *R*^2^ values. The secondary creep of PLA was probably caused by aging of this biodegradable polymer. The PLA aging results in partial polymer degradation that is connected with chain scissions, lowering of average molecular weight, and an increase of creep rate, as documented elsewhere [36,37].

The phenomenological EVP models accurately captured the initial creep stage observed in the *ε*–*t* curves but generally overestimated the creep strain at longer times due to the viscoous component of the model (the single dashpot element, occurring in both Equations (4) and (5) as a linear function of time). The same effect was described in previous studies [4,5]. With the increasing number of KV elements, the predictive accuracy of the EVP models somewhat improved, as evidenced by the higher *R*^2^ values and the reduced overestimation of long-term strain. As in the case of microindentation creep, we improved the stability for EVP models by fixing the retardation times (*τ*_i_ in Equation (4)) at suitable pre-fitted values. These pre-fitted values worked reasonably well for standard creep curves of PCL-rich blends, but failed in the case of PLA-rich blends, which exhibited low deformation, stepwise strain increments, and the secondary creep, as described in the previous paragraph. In the most extreme cases (Figure 5 and Appendix C), the *R*^2^ values were even negative, indicating that the EVP extrapolation failed completely and that more robust creep descriptors are needed, as discussed in the following section.

## 4. Discussion

### 4.1. Traditional Descriptors of Micro- and Macrocreep of Polymer Systems

Creep behavior is commonly characterized using the standardized indentation creep parameter, *C_IT_*, (ISO 14577 [22]; Equation (1)) and the creep exponent, *n*, obtained from the widely used empirical PL model (Equations (2) and (3) for macro- and microscale creep, respectively). Our previous study [5] demonstrated that these two parameters (*C_IT_* and *n*) could be employed to predict qualitatively the macroscale tensile creep behavior from the short-term microindentation creep measurements in the case of *homopolymers*. Therefore, we tried to apply the same creep descriptors to the PLA/PCL *polymer blends*.

The straightforward characterization of microcreep behavior of PLA/PCL blends with *C_IT_* failed completely, as already exemplified above in Section 3.1.2. Figure 3g suggested that the creep of PLA/PCL blends increased with increasing concentration of the stiffer PLA component, which is not correct or physically meaningful. Historically, the indentation creep parameter, *C_IT_*, was defined in ISO 14577 [22] to describe the behavior of engineering materials such as metals and ceramics. Consequently, its application to more complex elasto-visco-plastic systems, such as polymer blends, can yield misleading results. More detailed justification for the specific case of PLA/PCL blends is given in Appendix A.

**Figure 6:**
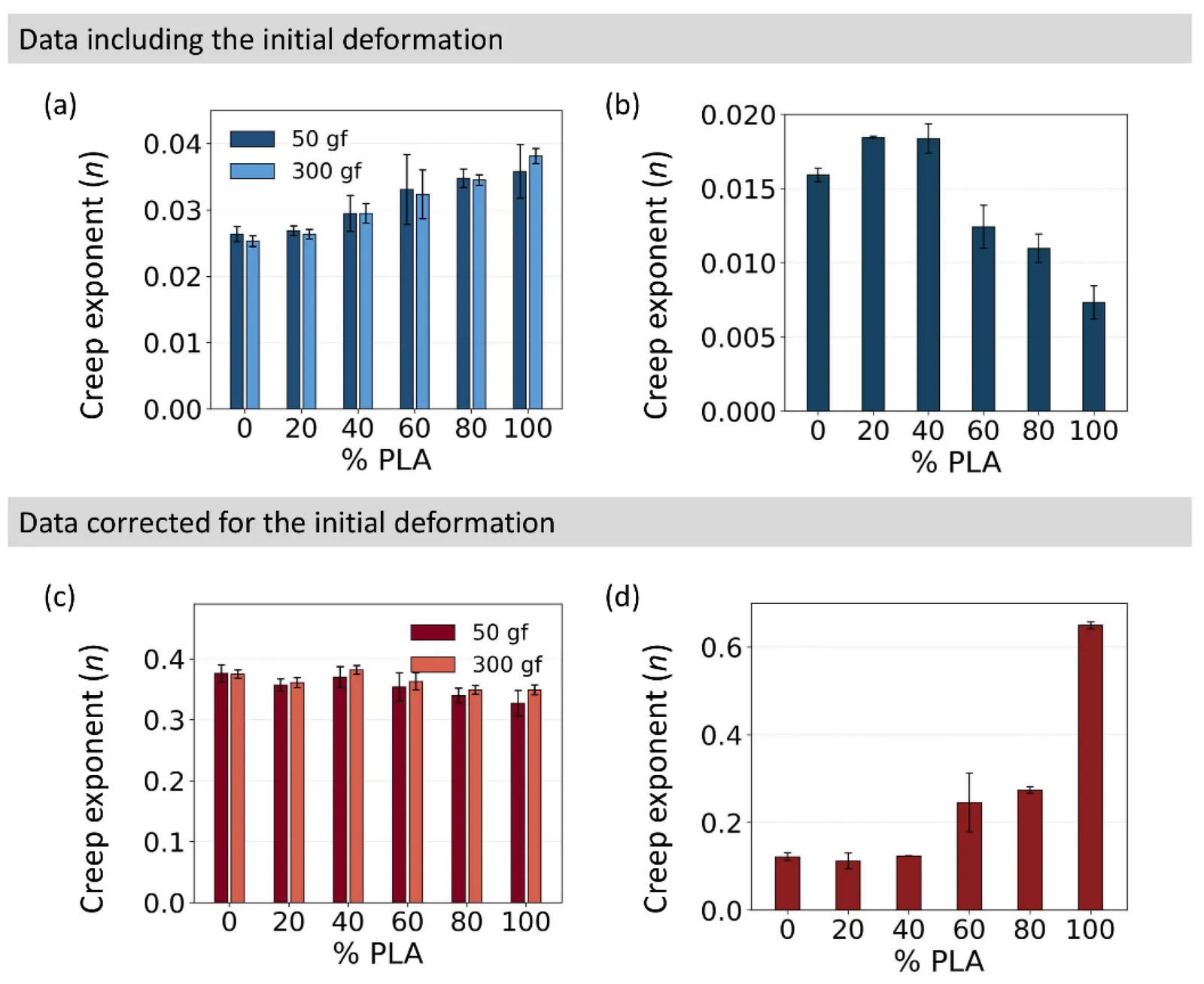
Power law exponent *n* obtained from fitting of PL to microscale indentation creep (left) and macroscale tensile creep (right). The top row (**a**,**b**) and bottom row (**c**,**d**) show the exponent before and after the data were corrected for the initial deformation, respectively. For tensile tests, the loading is immediate and the initial deformation is elastic, whereas for indentation tests, the loading takes a finite time and the initial deformation is elasto-visco-plastic.

The characterization of creep behavior with the PL exponent, *n*, is more universal in the sense that it can be applied not only to microscale indentation creep (like *C*_IT_) but also to macroscale tensile creep. The results are summarized in Figure 6. The creep exponent *n* was obtained from fitting the PL model to the original creep data (Figure 6a,b) and the data after subtraction of the initial deformation (Figure 6c,d). The term *initial deformation* refers to the deformation of the sample before the full loading is reached. For tensile tests, the loading is immediate (release of the dead weight) and the initial deformation is elastic, whereas for indentation tests, the loading takes a finite time (linear loading up to the maximum force) and the initial deformation is elasto-visco-plastic. If fitting was applied to the original data, incorrect trends were obtained, since *n* did not reflect the expected monotonic stiffening effect of the PLA component; microscale indentation creep (Figure 6a) showed misleading increase in creep with PLA concentration, as in the case of *C*_IT_ (Figure 3g). Macroscale tensile creep (Figure 6b) exhibited an illogical maximum around PLA/PCL-80/20 composition. Therefore, the initial deformation was subtracted before calculating the *n* exponent, allowing the analysis to focus exclusively on the subsequent elasto-visco-plastic creep deformation. For micromechanical data, the initial indentation depth (*h*_0_) was subtracted, while for macromechanical data, the instantaneous strain (*ε*_0_) was removed as described elsewhere [38]. After this correction, the *n* values obtained from the microscale indentation creep measurements showed a more consistent relationship with the expected mechanical behavior of the blends (Figure 6c): the increasing concentration of the stiffer PLA component resulted in a small but detectable progressive decrease in the *n* exponent. On the other hand, for the macroscale tensile creep measurements (Figure 6d), the exponent *n* increased with increasing PLA content (Figure 6d), which was connected with the secondary creep behavior of PLA-rich blends during the tensile testing, as documented in the next section.

In summary, neither of the two common creep descriptors could describe the observed decrease in creep with increasing PLA concentration. The ISO-defined *C*_IT_ exhibited the counter-intuitive increase with PLA concentration. The PL-based creep exponent *n* displayed varying trends, and the correction of the experimental data for the initial deformation slightly rectified the situation only for the indentation creep.

### 4.2. Alternative Descriptors of Micro- and Macrocreep in Polymer Systems

**Figure 7:**
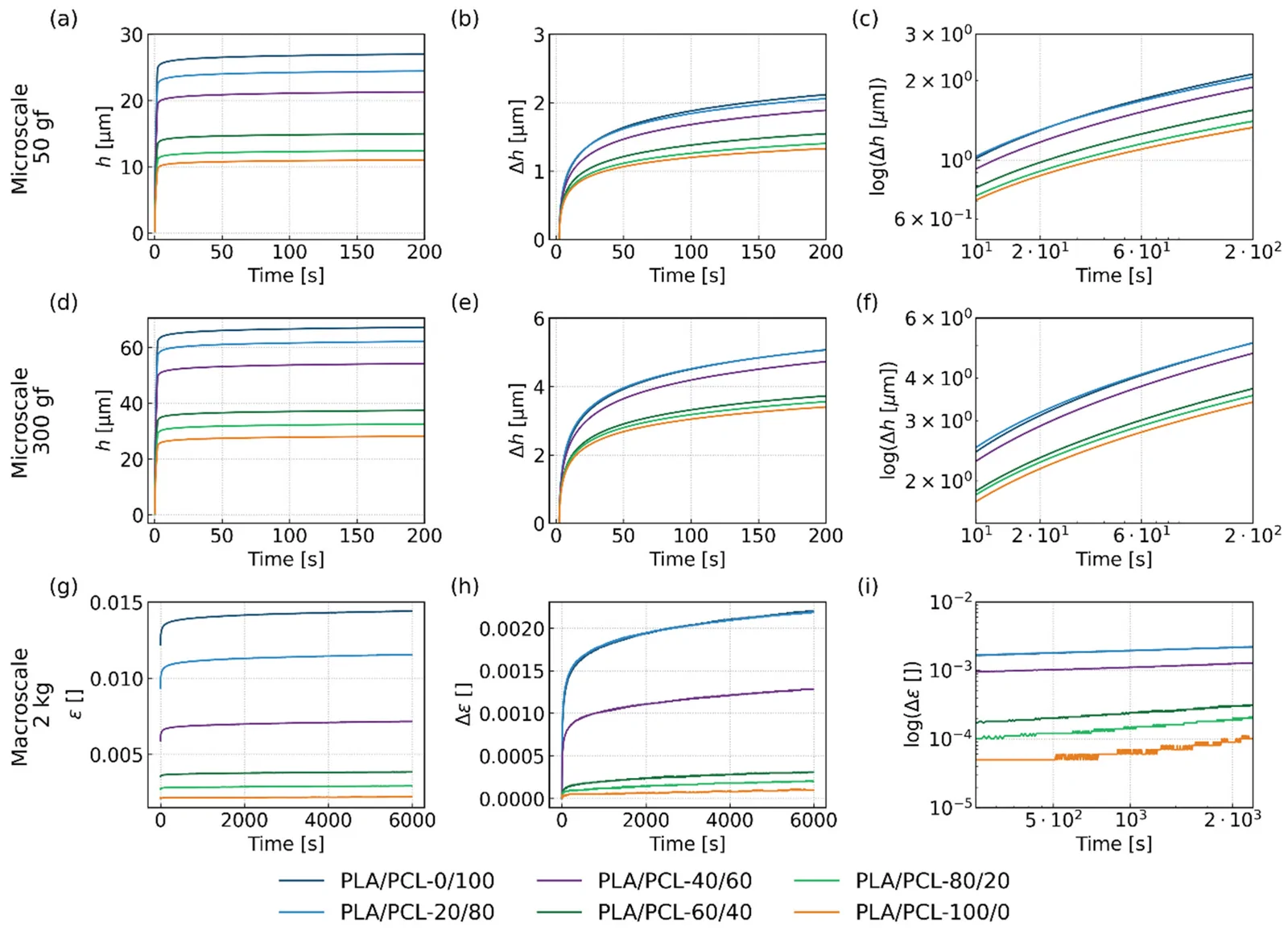
Average creep curves for (**a**–**c**) microindentation creep at 50 gf, (**d**–**f**) microindentation creep at 300 gf, and (**g**–**i**) macroscale tensile creep at 2 kg. All columns show deformation as a function of time. The left column displays total deformation (penetration depth *h* and strain *ε* for indentation and tensile creep, respectively). The middle column shows the deformation corrected for the initial deformation (*Δh* and *Δε* for indentation and tensile creep, respectively). The right column shows *Δh* and *Δε* as a function of time in log–log scale.

As the traditional creep descriptors failed to characterize the creep behavior of PLA/PCL blends correctly, we focused our attention on the analysis of the raw experimental data. Deformation from the indentation creep experiments (penetration depth, *h*) and tensile creep experiments (strain, *ε*) was plotted as a function of time (Figure 7). The objective was to explain the observed discrepancies and to search for alternative, more suitable creep descriptors.

The raw creep data without correction for the initial deformation (left column of Figure 7) revealed two key findings. First, the total creep deformation increased monotonically with increasing PCL concentration at all length scales. This suggested that parameters based on the total deformation could serve as suitable creep descriptors. Second, the initial deformation contributed substantially to the total deformation, thereby obscuring differences in the creep rate reflected by the creep exponent, *n*. Therefore, subtracting the initial deformation could facilitate further data analysis.

The creep data after subtraction of the initial deformation (middle column of Figure 7; the subtraction of initial deformation is explained in the previous section) retained the original sequence of the PLA/PCL blends, in which the total deformation increased with increasing PCL concentration. Moreover, the increase in creep rate with increasing PCL concentration became more evident. This explained the improved behavior of the microscale indentation creep exponent, *n*, after the background subtraction, as described in the previous section (cf. Figure 6a,c). However, it did not explain why the same improvement was not observed for the macroscale tensile creep exponent. The reason becomes clearer by taking the logarithm of the equation describing macroscale tensile creep (Equation (2)) after subtraction of the initial deformation, i.e., after replacing ε with Δε:

(6) $$\log\Delta \varepsilon \left(t\right)=\log C+\;n\cdot \log t$$

The modified equation shows that, within the PL model, the creep deformation should increase linearly in a log(*t*)–log(Δε) plot. Consequently, the log–log plots should reveal why the description based on the PL creep exponent fails. The log–log plots for microscale indentation creep and macroscale tensile creep after subtraction of the initial deformation are shown in the right column of Figure 7. For microscale indentation creep (Figure 7c,f), the average slope of the curves, represented by the PL creep exponent, *n*, increased moderately with increasing PCL concentration, although the creep curves were not perfectly linear and the increase in *n* was relatively small. In contrast, for macroscale tensile creep (Figure 7i), the average slope of the curves for the PLA-rich blends was strongly affected by the secondary creep of PLA, which increased the *n* values despite the lower total deformation compared with the PCL-rich blends. It is worth noting that the primary creep rate is the lowest and gradually decreases with time, and the secondary creep rate is higher and remains approximately constant, whereas the tertiary creep rate is the highest, increases with time, and eventually leads to specimen failure [39]. The occurrence of secondary creep in PLA under tensile loading is not uncommon, as reported by Yi-Sheng Jhao et al. [40].

In summary, the analysis of the micro- and macroscale creep curves (Figure 7) demonstrated that the total deformation was strongly influenced by the initial deformation and that the creep exponent, *n*, was not a suitable descriptor of the creep behavior of PLA/PCL blends. For microscale indentation creep, *n* exhibited a weak but correct trend after correction for the initial deformation. In contrast, for macroscale tensile creep, *n* was too sensitive to the secondary creep of PLA. Conversely, the parameters *h*, *ε*, *Δh*, and *Δε*, which describe the total creep deformation, exhibited consistent trends at both length scales. Their systematic evolution from PCL-rich to PLA-rich blends suggested that they were less sensitive to scale-dependent deformation mechanisms than the creep exponent, *n*. Therefore, they emerged as promising descriptors for establishing predictive correlations between microscale indentation creep and macroscale tensile creep in PLA/PCL blends.

### 4.3. Representative Parameters to Describe Creep Behavior in PLA/PCL Blends

**Figure 8:**
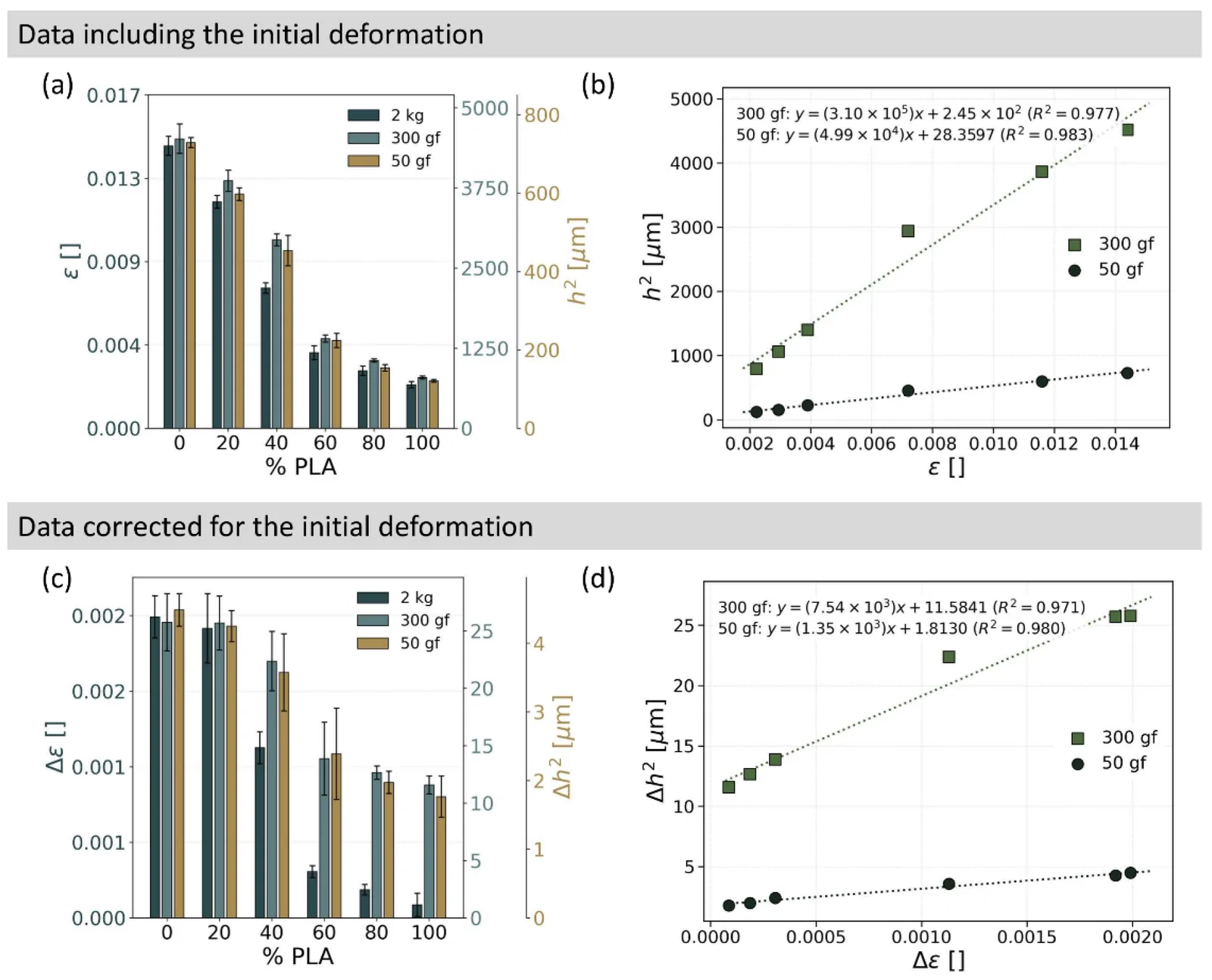
Final creep descriptors suitable for the characterization of PLA/PCL blends: (**a**) total deformations *ε* and *h*^2^ obtained from tensile and indentation experiments, respectively, (**b**) *ε*–*h*^2^ correlation, (**c**) total deformations *Δh*^2^ and *Δε* after subtraction of initial deformation, and (**d**) Δ*ε*–Δ*h*^2^ correlation. For tensile tests, the loading is immediate and the initial deformation is elastic, whereas for indentation tests, the loading takes a finite time and the initial deformation is elasto-visco-plastic. The correlation plots (**b**,**d**) also show linear regression curves, regression equations, and coefficients of determination (*R*^2^).

Figure 8 summarizes the total deformation parameters (*h*, *ε*, *Δh*, and *Δε*) obtained from the microscale indentation creep and macroscale tensile creep measurements. The total deformation parameters were identified in the previous section as descriptors enabling consistent characterization of the creep behavior of PLA/PCL blends. These parameters show not only the same trends (Figure 8a,c) but also strong linear correlations (Figure 8b,d; all *R*^2^ > 0.97).

It is worth noting that Figure 8 compares the tensile strain before and after correction for the initial deformation (*ε* and *Δε*) with the physically equivalent quantities obtained from indentation experiments, namely the square of the penetration depth before and after correction for the initial deformation (*h*^2^ and *Δh*^2^). For tensile creep experiments, the loading (i.e., the release of dead weight) is immediate and the initial deformation is elastic, while for the indentation tests, the loading takes non-zero time (linear loading up to maximum force) and, as a result, the initial deformation is elasto-visco-plastic. The reason why *ε* is equivalent to *h*^2^ is best illustrated by the equations relating strain and penetration depth to creep compliance, *C*(*t*):

(7) $$\varepsilon \left(t\right)=\sigma \cdot C(t)$$

(8) $$h{\left(t\right)}^{2}=KP\cdot C(t)$$

where Equation (7) represents the general relationship between tensile stress and strain, whereas Equation (8) relates the penetration depth to creep compliance for cylindrical, spherical, and conical indenters [41]. In Equation (8), *K* is a geometric constant depending on the indenter shape and Poisson’s ratio, and *P* is the applied load [4,41]. It is worth noting that the exact form of *K* is also influenced by the definition of *C*(*t*), which may be formulated for tensile or shear experiments with or without incorporating specific values of Poisson’s ratio [4,5,41,42].

**Figure 9:**
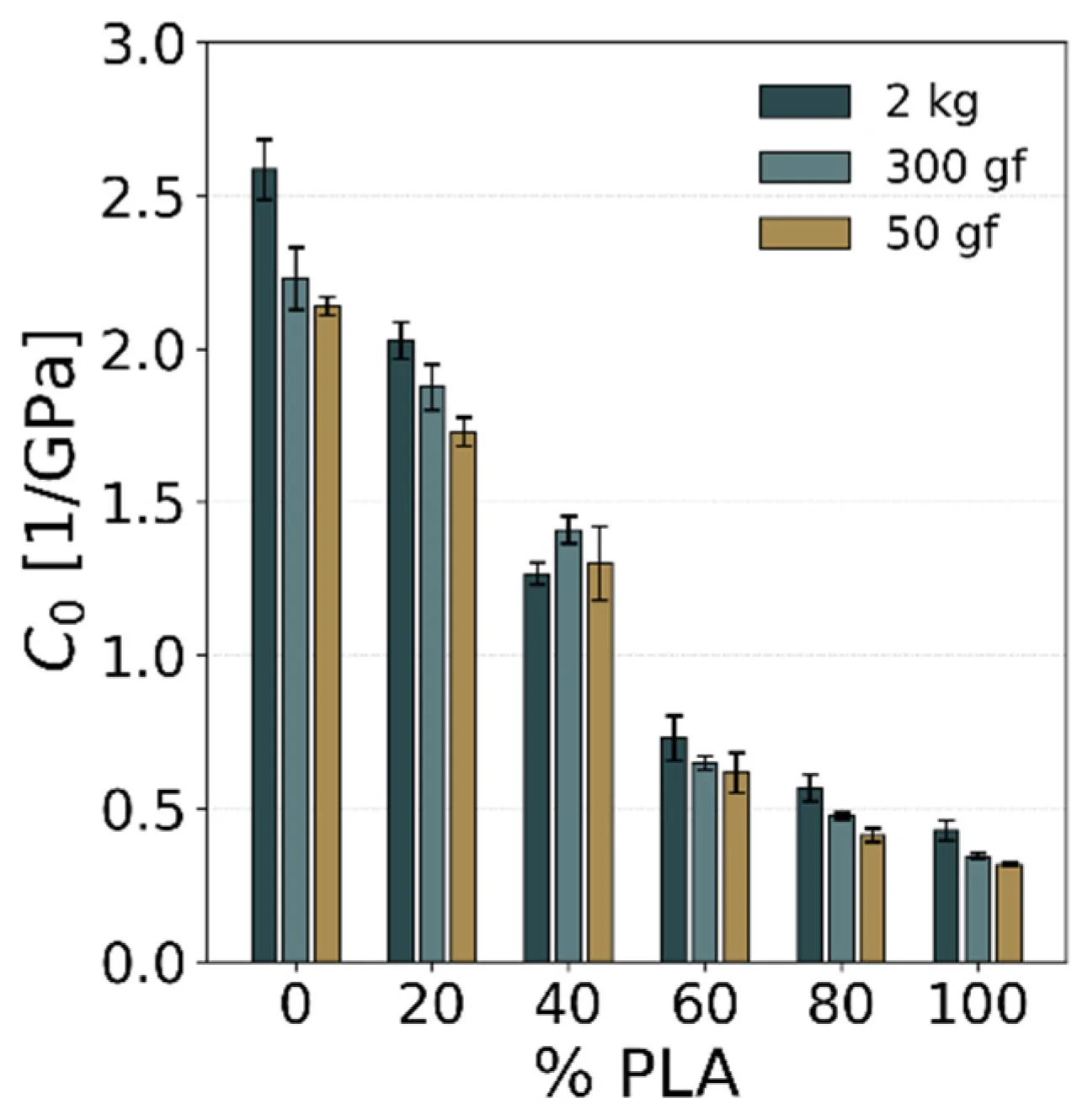
Creep compliance, *C*_0_, obtained by fitting the EVP3 model to macroscale tensile creep data (loading: 2 kg) and microscale indentation creep data (loadings: 50 and 300 gf).

Figure 9 displays the initial compliance, *C*_0_, which is an additional parameter that could be extracted from fitting of EVP models (Equations (4) and (5)) to creep data with our MCREEP program package, as described in our previous work and references therein [5]. The compliance is the reciprocal of the elastic modulus for a given loading mode; in our case, the initial compliance obtained from EVP fitting corresponds to *C*_0_ = 1/*E*_0_, where *E*_0_ is the initial tensile or indentation modulus from macroscale or microscale creep experiments, respectively. The elastic moduli estimated from the creep experiments (*E*_0_ = 1/*C*_0_; Figure 9) were in very good agreement with the elastic moduli determined from the independent tensile tests (*E* in Figure 3a) and with the indentation moduli evaluated using the Oliver–Pharr method (*E*_IT_ in Figure 3a). This agreement further supports the reliability of both our experimental measurements (tensile tests vs. creep tests) and data processing (fitting of creep data using the EVP models).

The key conclusion is that the final creep descriptors shown in Figure 8 exhibit the same trends (Figure 8a,c) and strong linear correlations (Figure 8b,d) between microscale indentation creep and macroscale tensile creep. Therefore, the total deformation parameters from short-term microscale indentation creep (*h*^2^ and Δ*h*^2^) can qualitatively predict the macroscale creep deformation (*ε* and *Δε*) for PLA/PCL blends. Importantly, these results remain consistent over the entire concentration range, from soft and ductile PCL-rich blends to stiff and brittle PLA-rich blends. This suggests that these parameters might also be applicable to other polymer systems; verification of this assumption is the subject of our ongoing research. Furthermore, the instantaneous compliances extracted from EVP modeling of indentation and tensile creep are quantitatively comparable with each other and with literature values, further confirming the correctness and reliability of the proposed approach.

## 5. Conclusions

We compared microscale indentation creep with macroscale tensile creep in immiscible PLA/PCL blends. Microscale creep was characterized by instrumented indentation at two applied loads (50 and 300 gf), and macroscale creep by tensile experiments at a single load (2 kg). Various creep descriptors and models, comprising an empirical power law model and three phenomenological elasto-visco-plastic models, were employed to analyze the creep response at both length scales. The main findings can be summarized as follows:

- Microindentation measurements reproduced the trends observed in tensile creep experiments. Despite the different stress fields and deformation volumes involved, both techniques consistently ranked the investigated PLA/PCL blends according to their creep resistance, provided that appropriate creep descriptors were used.
- The conventional creep descriptors, namely the indentation creep parameter, *C*_IT_ (defined in the ISO standard), and the creep exponent, *n,* from the widely used empirical power law model, did not provide reliable micro-to-macro creep correlations for the investigated PLA/PCL blends. The indentation creep parameter *C*_IT_ did not adequately capture the complex evolution of elasto-visco-plastic behavior across the investigated systems, ranging from very soft and ductile PCL-rich blends to very stiff and brittle PLA-rich blends. The creep exponent *n* was strongly affected by PLA aging, which promoted the development of secondary creep during tensile loading.
- In contrast, the total creep deformation parameters, such as the squared penetration depth from microindentation (Δ*h*^2^) and the tensile strain from macroscale creep measurements (Δ*ε*), both corrected for the initial deformation, showed consistent trends across all investigated compositions and testing conditions and exhibited strong linear correlations (*R*^2^ > 0.97).
- These results demonstrate that short-term microindentation creep measurements can correctly rank polymer blends according to their macroscale tensile creep behavior, provided that suitable creep descriptors are employed. Considering that the PLA/PCL blends cover a broad range of properties, from soft and ductile PCL-rich compositions to stiff and brittle PLA-rich compositions, the results suggest that total deformation parameters might also serve as reliable creep descriptors for other polymer systems, including copolymers, crosslinked polymers, and composites.

## Figures and Tables

**Table 1:** Summary of the prepared blends.

Samples	PLA [%]	PCL [%]
PLA/PCL-0/100	0	100
PLA/PCL-20/80	20	80
PLA/PCL-40/60	40	60
PLA/PCL-60/40	60	40
PLA/PCL-80/20	80	20
PLA/PCL-100/0	100	0

**Figure A1:**
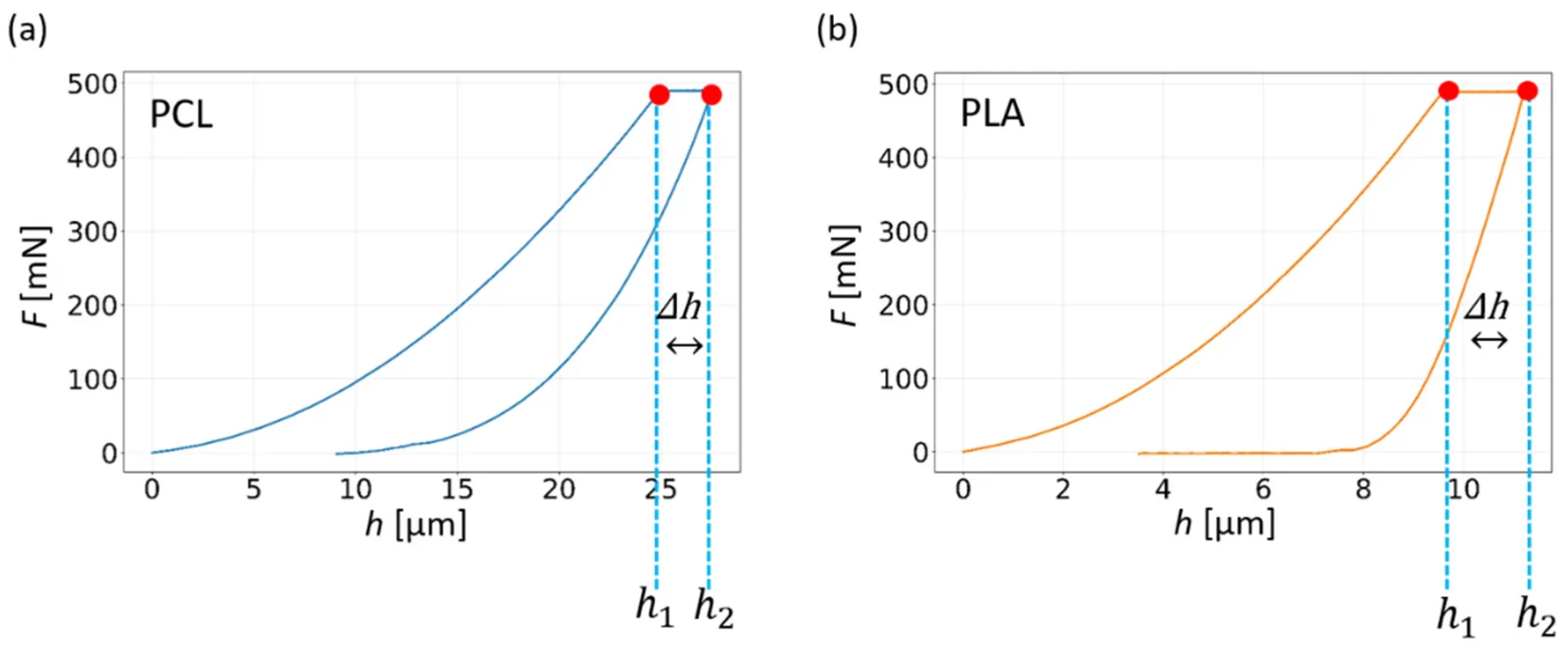
Explanation of *C*_IT_ and *η*_IT_ behavior of the two components of the PLA/PCL blends—representative *F*–*h* curves of (**a**) pure PCL and (**b**) pure PLA.

## Data Availability

The original contributions presented in this study are included in the article. Further inquiries can be directed to the corresponding author.

## Appendix A. *C_IT_* and *η*_IT_ Behavior in PLA/PCL Polymer Blends

This appendix summarizes the behavior of *C*_IT_ and *η*_IT_ for the polymer blends. Figure A1 presents representative *F*–*h* curves from microscale indentation creep measurements for the pure PCL and PLA. The *F*–*h* curves illustrate the origin of the apparently counterintuitive trends observed for both parameters.

The initial indentation depth (*h*_1_) is lower for the pure PLA due to its higher stiffness. According to Equation (1), *C*_IT_ is calculated relative to this indentation depth. Consequently, although PLA exhibits a smaller creep deformation, its normalized indentation creep value can be higher because the creep displacement is divided by a smaller initial indentation depth. Therefore, the higher *C*_IT_ values obtained for PLA-rich blends arise from the normalization procedure used in the definition of this parameter rather than from a greater intrinsic creep susceptibility.

A similar explanation applies to *η*_IT_. According to ISO 14577 [22], *η*_IT_ is defined as the ratio between the elastic work recovered during unloading (*W_elast_*) and the total indentation work (*W_tota_*_l_). Although PLA exhibits higher stiffness and a smaller maximum indentation depth than PCL, its unloading curve encloses a smaller area, resulting in a lower recovered elastic work. In contrast, the larger indentation depth of PCL increases the area under the unloading curve and, consequently, the recovered elastic work. Therefore, the higher *η_IT_* values obtained for PCL-rich blends originate from the geometry of the loading–unloading curves used in the calculation of *η*_IT_ rather than indicating that PCL is intrinsically more elastic than PLA.

The indentation creep parameters *C*_IT_ and *η*_IT_ defined in ISO 14577 [22] were originally developed as phenomenological descriptors for instrumented indentation of engineering materials, particularly metals and ceramics, in which time-dependent deformation is generally limited. When applied to polymers, whose mechanical response is governed by complex viscoelastic and viscoplastic mechanisms, these parameters should not be interpreted as intrinsic measures of viscosity or elasticity. Instead, they reflect the combined contributions of elastic, viscoelastic, and plastic deformation together with the evolving contact geometry during indentation. Consequently, the ranking of polymers based on *C*_IT_ or *η*_IT_ may differ from that obtained using conventional rheological measurements or macroscopic creep tests.

## Appendix B. Fitting of Creep Models to Microindentation Creep Data

This appendix summarizes the fitting results of the four creep models applied to the short-term microscale indentation creep data (hold time = 200 s, loading 50 gf and 300 gf). The averaged fitting parameters of the PL model are presented in Table A1, while the corresponding results for the EVP models are given in Table A2 and Table A3. The reported values represent the average of all measurements and model fittings performed for each sample/load combination (>15 measurements). The EVP and PL models, together with their fitting parameters, are described elsewhere [4,5]. For the EVP2 and EVP3 models, the retardation times (*τ_i_*) were fixed at suitable pre-fitted values to improve the stability of the fit as described elsewhere [4,5]. The coefficients of determination, $R_{fit}^{2}$ and $R_{all}^{2}$, correspond to the fitted data and the complete experimental dataset, respectively. The coefficient of determination takes the value of 1.00 for perfect fit and a lower value otherwise [43]. Since the models were fitted over the entire experimental time range, the coefficients are identical ($R_{fit}^{2}=R_{all}^{2}$).

**Table A1:** Parameters from the fitting of PL model to microindentation creep (hold time 200 s, loading 50 gf and 300 gf).

Sample	Model	Load (gf)	*C*	*n*	$R_{fit}^{2}$	$R_{all}^{2}$
PLA/PCL-0/100	PL ^1^	50	1126.98	0.02637	0.9992	0.9866
PLA/PCL-20/80	PL	50	921.46	0.02688	0.9991	0.9822
PLA/PCL-40/60	PL	50	691.08	0.02950	0.9991	0.9865
PLA/PCL-60/40	PL	50	335.43	0.03310	0.9990	0.9825
PLA/PCL-80/20	PL	50	228.37	0.03476	0.9990	0.9788
PLA/PCL-100/0	PL	50	178.41	0.03582	0.9983	0.9716
PLA/PCL-0/100	PL	300	7029.10	0.02532	0.9995	0.9868
PLA/PCL-20/80	PL	300	5975.95	0.02636	0.9995	0.9833
PLA/PCL-40/60	PL	300	4473.36	0.02950	0.9997	0.9894
PLA/PCL-60/40	PL	300	2098.78	0.03235	0.9997	0.9856
PLA/PCL-80/20	PL	300	1569.35	0.03450	0.9996	0.9820
PLA/PCL-100/0	PL	300	1151.76	0.03815	0.9994	0.9808

^1^ PL = power law model.

**Table A2:** Final parameters from the fitting of EVP models to short-term indentation creep (hold time 200 s, loading 50 gf).

Sample	Model ^1^	$C_{0}$ (GPa^−1^)	$C_{\upsilon }$ (GPa^−1^ s^−1^)	$C_{1}$ (GPa^−1^)	$C_{2}$ (GPa^−1^)	$C_{3}$ (GPa^−1^)	$\tau_{1}$ (s)	$\tau_{2}$ (s)	$\tau_{3}$ (s)	$R_{fit}^{2}$	$R_{all}^{2}$
PLA/PCL-0/100	EVP1	2.2709	0.00056	0.2157	x	x	15.4	x	x	0.9909	0.9909
PLA/PCL-20/80	EVP1	1.8474	0.00048	0.1886	x	x	14.6	x	x	0.9898	0.9898
PLA/PCL-40/60	EVP1	1.3941	0.00039	0.1479	x	x	15.3	x	x	0.9901	0.9901
PLA/PCL-60/40	EVP1	0.6732	0.00022	0.0838	x	x	14.4	x	x	0.9890	0.9890
PLA/PCL-80/20	EVP1	0.4557	0.00016	0.0634	x	x	14.0	x	x	0.9884	0.9884
PLA/PCL-100/0	EVP1	0.3531	0.00013	0.0541	x	x	13.5	x	x	0.9877	0.9877
PLA/PCL-0/100	EVP2	2.1629	0.00042	0.1911	0.1564	x	3	30	x	0.9994	0.9994
PLA/PCL-20/80	EVP2	1.7507	0.00035	0.1747	0.1318	x	3	30	x	0.9993	0.9993
PLA/PCL-40/60	EVP2	1.3182	0.00029	0.1334	0.1065	x	3	30	x	0.9993	0.9993
PLA/PCL-60/40	EVP2	0.6292	0.00016	0.0794	0.0578	x	3	30	x	0.9992	0.9992
PLA/PCL-80/20	EVP2	0.4223	0.00012	0.0610	0.0430	x	3	30	x	0.9991	0.9991
PLA/PCL-100/0	EVP2	0.3247	0.00009	0.0529	0.0357	x	3	30	x	0.9991	0.9991
PLA/PCL-0/100	EVP3	2.1427	0.00030	0.1608	0.1019	0.1282	2	10	50	0.9998	0.9998
PLA/PCL-20/80	EVP3	1.7305	0.00025	0.1503	0.0883	0.1073	2	10	50	0.9997	0.9997
PLA/PCL-40/60	EVP3	1.3003	0.00021	0.1187	0.0662	0.0896	2	10	50	0.9997	0.9997
PLA/PCL-60/40	EVP3	0.6178	0.00012	0.0723	0.0371	0.0483	2	10	50	0.9996	0.9996
PLA/PCL-80/20	EVP3	0.4135	0.00008	0.0555	0.0282	0.0356	2	10	50	0.9996	0.9996
PLA/PCL-100/0	EVP3	0.3176	0.00007	0.0475	0.0244	0.0291	2	10	50	0.9996	0.9996

^1^ Models: EVP1 = S + D + 1 KV, EVP2 = S + D + 2 KV, and EVP3 = S + D + 3 KV.

**Table A3:** Final parameters from the fitting of EVP models to short-term indentation creep (hold time 200 s, loading 300 gf).

Sample	Model ^1^	$C_{0}$ (GPa^−1^)	$C_{\upsilon }$ (GPa^−1^ s^−1^)	$C_{1}$ (GPa^−1^)	$C_{2}$ (GPa^−1^)	$C_{3}$ (GPa^−1^)	$\tau_{1}$ (s)	$\tau_{2}$ (s)	$\tau_{3}$ (s)	$R_{fit}^{2}$	$R_{all}^{2}$
PLA/PCL-0/100	EVP1	2.3602	0.00056	0.2150	x	x	15.4	x	x	0.9908	0.9908
PLA/PCL-20/80	EVP1	1.9991	0.00050	0.1977	x	x	14.7	x	x	0.9901	0.9901
PLA/PCL-40/60	EVP1	1.5081	0.00042	0.1559	x	x	15.6	x	x	0.9908	0.9908
PLA/PCL-60/40	EVP1	0.7044	0.00022	0.0841	x	x	14.8	x	x	0.9895	0.9895
PLA/PCL-80/20	EVP1	0.5239	0.00018	0.0702	x	x	14.2	x	x	0.9889	0.9889
PLA/PCL-100/0	EVP1	0.3840	0.00015	0.0581	x	x	14.4	x	x	0.9891	0.9891
PLA/PCL-0/100	EVP2	2.2518	0.00041	0.1910	0.1561	x	3	30	x	0.9995	0.9995
PLA/PCL-20/80	EVP2	1.8977	0.00037	0.1821	0.1392	x	3	30	x	0.9994	0.9994
PLA/PCL-40/60	EVP2	1.4283	0.00032	0.1391	0.1136	x	3	30	x	0.9995	0.9995
PLA/PCL-60/40	EVP2	0.6602	0.00017	0.0787	0.0591	x	3	30	x	0.9994	0.9994
PLA/PCL-80/20	EVP2	0.4867	0.00013	0.0673	0.0480	x	3	30	x	0.9993	0.9993
PLA/PCL-100/0	EVP2	0.3535	0.00011	0.0550	0.0401	x	3	30	x	0.9994	0.9994
PLA/PCL-0/100	EVP3	2.2307	0.00030	0.1622	0.1008	0.1286	2	10	50	0.9999	0.9999
PLA/PCL-20/80	EVP3	1.8769	0.00027	0.1562	0.0928	0.1135	2	10	50	0.9999	0.9999
PLA/PCL-40/60	EVP3	1.4103	0.00023	0.1227	0.0704	0.0955	2	10	50	0.9999	0.9999
PLA/PCL-60/40	EVP3	0.6487	0.00012	0.0718	0.0370	0.0498	2	10	50	0.9998	0.9998
PLA/PCL-80/20	EVP3	0.4768	0.00010	0.0616	0.0310	0.0400	2	10	50	0.9998	0.9998
PLA/PCL-100/0	EVP3	0.3458	0.00008	0.0496	0.0260	0.0333	2	10	50	0.9998	0.9998

^1^ Models: EVP1 = S + D + 1 KV, EVP2 = S + D + 2 KV, and EVP3 = S + D + 3 KV.

## Appendix C. Fitting of Creep Models to Macro Tensile Creep Data

This appendix summarizes the results obtained by fitting the four investigated creep models to the macro tensile creep data. The fitting parameters of the PL model are presented in Table A4, while the corresponding results for the EVP models are given in Table A5. The reported values represent averages from all experimental measurements and corresponding model fittings, with each sample/load combination measured at least twice.

The retardation times (*τ_i_*) for the EVP1, EVP2, and EVP3 models were fixed at suitable values to enhance the stability of the fit, as described elsewhere [4,5]. The coefficients of determination, $R_{fit}^{2}$ and $R_{all}^{2}$, were calculated for the fitted data (up to 1000 s) and all data (up to 6000 s), respectively. As discussed above, this approach was adopted to evaluate the long-term predictive capability of the models by comparing their predictions with the complete experimental creep response. Like in Appendix B, the *R*^2^(fit) values were close to unity, indicating that all models were able to adequately describe the short-term creep behavior used for fitting. However, the *R*^2^(all) values decreased when the complete creep curves were considered, demonstrating the increased difficulty of accurately predicting the long-term creep response.

**Table A4:** Parameters from the fitting of PL model to macro tensile creep (hold time 6000 s, loading 2 kg).

Sample	Model	*C*	*n*	$R_{fit}^{2}$	$R_{all}^{2}$
PLA/PCL-0/100	PL ^1^	0.0125	0.0168	0.9944	0.9846
PLA/PCL-20/80	PL	0.0098	0.0192	0.9938	0.9870
PLA/PCL-40/60	PL	0.0061	0.0177	0.9924	0.9896
PLA/PCL-60/40	PL	0.0035	0.0086	0.9872	0.6524
PLA/PCL-80/20	PL	0.0028	0.0053	0.9220	0.0930
PLA/PCL-100/0	PL	0.0021	0.0034	0.8153	0.1476

^1^ PL = power law model.

**Table A5:** Final parameters from the fitting of EVP models to macro tensile creep (hold time 6000 s, load 2 kg).

Sample	Model ^1^	$C_{0}$ (GPa^−1^)	$C_{\upsilon }$ ^2^ (GPa^−1^s^−1^)	$C_{1}$ (GPa^−1^)	$C_{2}$ (GPa^−1^)	$C_{3}$ (GPa^−1^)	$\tau_{1}$ (s)	$\tau_{2}$ (s)	$\tau_{3}$ (s)	$R_{fit}^{2}$	$R_{all}^{2}$
PLA/PCL-0/100	EVP1	2.6493	0.000056	0.1491	x	x	100	x	x	0.9908	−1.8397
PLA/PCL-20/80	EVP1	2.0876	0.000055	0.1349	x	x	100	x	x	0.9920	−3.4665
PLA/PCL-40/60	EVP1	1.3001	0.000029	0.0784	x	x	100	x	x	0.9886	−1.0582
PLA/PCL-60/40	EVP1	0.7367	0.000011	0.0170	x	x	100	x	x	0.9835	−1.1220
PLA/PCL-80/20	EVP1	0.5696	0.000006	0.0071	x	x	100	x	x	0.9266	0.2376
PLA/PCL-100/0	EVP1	0.4352	0.000001	0.0079	x	x	100	x	x	0.9482	−3.0866
PLA/PCL-0/100	EVP2	2.5643	0.000035	0.1420	0.1117	x	20	200	x	0.9989	0.4825
PLA/PCL-20/80	EVP2	2.0052	0.000034	0.1355	0.1006	x	20	200	x	0.9989	0.1498
PLA/PCL-40/60	EVP2	1.2533	0.000019	0.0788	0.0560	x	20	200	x	0.9989	0.6722
PLA/PCL-60/40	EVP2	0.7272	0.000009	0.0161	0.0126	x	20	200	x	0.9886	0.2162
PLA/PCL-80/20	EVP2	0.5625	0.000007	0.0118	0.0020	x	20	200	x	0.9405	−0.3163
PLA/PCL-100/0	EVP2	0.4343	0.000001	0.0014	0.0073	x	20	200	x	0.9495	−2.7069
PLA/PCL-0/100	EVP3	2.5438	0.000031	0.0966	0.1016	0.0832	10	50	300	0.9998	0.6653
PLA/PCL-20/80	EVP3	1.9848	0.000028	0.0951	0.0908	0.0794	10	50	300	0.9996	0.6206
PLA/PCL-40/60	EVP3	1.2354	0.000015	0.0634	0.0498	0.0446	10	50	300	0.9991	0.9057
PLA/PCL-60/40	EVP3	0.7226	0.000008	0.0140	0.0103	0.0103	10	50	300	0.9892	0.5128
PLA/PCL-80/20	EVP3	0.5638	0.000009	0.0040	0.0093	−0.0027	10	50	300	0.9467	−2.7072
PLA/PCL-100/0	EVP3	0.4373	−0.000001	−0.0042	0.0072	0.0029	10	50	200	0.9482	−3.3613

^1^ Models: EVP1 = S + D + 1 KV, EVP2 = S + D + 2 KV, and EVP3 = S + D + 3 KV. ^2^ *C*_v_ = *σ*/*η* as defined in Equation (4).
